# Antidepressants Impact Connexin 43 Channel Functions in Astrocytes

**DOI:** 10.3389/fncel.2015.00495

**Published:** 2016-01-07

**Authors:** Tiffany Jeanson, Audrey Pondaven, Pascal Ezan, Franck Mouthon, Mathieu Charvériat, Christian Giaume

**Affiliations:** ^1^Collège de France, Center for Interdisciplinary Research in Biology/Centre National de la Recherche Scientifique, Unité Mixte de Recherche 7241/Institut National de la Santé et de la Recherche Médicale U1050Paris, France; ^2^University Pierre et Marie CurieParis, France; ^3^MemoLife Laboratory of Excellence and Paris Science Lettre Research UniversityParis, France; ^4^TheranexusLyon, France

**Keywords:** gap Junctions, hemichannels, glial cells, depression, inflammation

## Abstract

Glial cells, and in particular astrocytes, are crucial to maintain neuronal microenvironment by regulating energy metabolism, neurotransmitter uptake, gliotransmission, and synaptic development. Moreover, a typical feature of astrocytes is their high expression level of connexins, a family of membrane proteins that form gap junction channels allowing intercellular exchanges and hemichannels that provide release and uptake pathways for neuroactive molecules. Interestingly, several studies have revealed unexpected changes in astrocytes from depressive patients and rodent models of depressive-like behavior. Moreover, changes in the expression level of the astroglial connexin 43 (Cx43) have been reported in a depressive context. On the other hand, antidepressive drugs have also been shown to impact the expression of this connexin in astrocytes. However, so far there is little information concerning the functional consequence of these changes, i.e., the status of gap junctional communication and hemichannel activity in astrocytes exposed to antidepressants. In the present work we focused our attention on the action of seven antidepressants from four different therapeutic classes and tested their effects on Cx43 expression and on the two connexin-based channels functions studied in cultured astrocytes. We here report that when used at non-toxic and clinically relevant concentrations they have no effects on Cx43 expression but differential effects on Cx43 gap junction channels. Moreover, all tested antidepressants inhibit Cx43 hemichannel with different efficiency depending on their therapeutic classe. By studying the impact of antidepressants on the functional status of astroglial connexin channels, contributing to dynamic neuroglial interactions, our observations should help to better understand the mechanism by which these drugs provide their effect in the brain.

## Introduction

During the two last decades, a major step in the understanding of brain functions and dysfunctions has been to consider that not only neurons are at the center of these processes but that also their glial environment is actively involved. This statement is particularly true for astrocytes, a major glial cell population that establishes tight morphological and functional interactions with neurons (Halassa and Haydon, 2010; Verkhratsky et al., 2012) leading to the concept of the “tripartite synapse” (see Araque et al., 1999; Pérez-Alvarez and Araque, 2013). In brain pathologies and mental illness this partnership is impaired, contributing to severe neuronal defects and even in certain cases leading to neuronal death (Giaume et al., 2007; Bennett et al., 2012; Parpura et al., 2012). Such alterations in neuroglial interaction start to be investigated in order to identify and develop alternative therapeutic approaches that target astrocytes instead of solely neurons (Colangelo et al., 2014; Lundgaard et al., 2014; Vardjan et al., 2015). Following this strategy, the objective is to act on a specific astroglial molecular constituent known to regulate neuronal activity and/or survival. Based on these requirements astroglial connexins (Cxs), a family of membrane proteins, may be considered as a good candidate. Indeed, Cxs are highly expressed in astrocytes compared to all other brain cell populations (see Ransom and Giaume, 2013), including neurons, and they have been reported to interplay with synaptic activity and plasticity (Pannasch et al., 2011), animal behavior (Stehberg et al., 2012), and neuronal survival (Froger et al., 2010; Freitas-Andrade and Naus, 2015). Besides, the expression and function of astroglial Cxs are affected in neurodegenerative diseases (Kawasaki et al., 2009; Koulakoff et al., 2012; Takeuchi and Suzumura, 2014), ischemia and stroke (Orellana et al., 2014), epilepsy (Mylvaganam et al., 2014), demyelinating diseases (Cotrina and Nedergaard, 2012) and cancer (Naus and Laird, 2010). Much less is known about the status, i.e., expression and function, of Cxs in astrocytes in non-neurodegenerative mood disorders such as depression, while those pathologies are associated to a reduction in the number of astrocytes and a decrease in GFAP immunoreactivity (see Rajkowska and Stockmeier, 2013).

Connexins are the molecular constituents of gap junctions that are membrane specializations consisting of dense aggregates of large pore channels formed by two paired hexamers of Cxs. These gap junction channels extend from one cell into an adjacent cell and mediate a unique direct cytoplasm-to-cytoplasm communication. These channels are poorly selective for ions and for small molecular weight signaling molecules, thus they allow extensive ionic and biochemical exchanges between cells (Harris, 2007). In astrocytes, gap junction channels provide the basis for ionic homeostasis, particularly for potassium buffering and intercellular calcium signaling. They are also involved in biochemical and metabolic coupling (see Ransom and Giaume, 2013). Under certain conditions Cxs can also operate as half of a gap junction channel, named “hemichannel,” representing another functional state that provides a pathway suitable for autocrine as well as paracrine interactions in the brain. In astrocytes, connexin hemichannels are permeable to ions and are involved in the release of gliotransmitters such as ATP and glutamate (Ye et al., 2003; Kang et al., 2008; Abudara et al., 2015), the uptake of glucose (Retamal et al., 2007) and the eﬄux of glutathione (Rana and Dringen, 2007; Ye et al., 2015). In astrocytes two major Cxs have been identified, Cx43 and Cx30, which are not expressed in other brain cell types and are characterized by different developmental and regional patterns of expression (Nagy et al., 2004). Both Cxs contribute to gap junctional communication but so far only hemichannels made by Cx43 have been reported to be functional in astrocytes (see Giaume et al., 2013).

The information concerning astroglial Cxs and depression is based on two kinds of observations available from the literature. Firstly, those that report changes in the expression of astroglial Cxs from depressive patients, persons having committed suicide or from animal models of depression. Indeed, a Canadian study of postmortem generated microarray data suicide completers indicated that the expression level of Cx43 and Cx30 is reduced in dorsal lateral prefrontal cortex (Ernst et al., 2011) and in the locus coeruleus (Bernard et al., 2011). In addition, a recent study has reported that the expression of Cx43 is reduced in postmortem brains from patients suffering from major depressive disorder or comorbid depression relative to healthy subjects (Miguel-Hidalgo et al., 2014). Also, Sun et al. (2012) have reported a decrease in diffusion of gap junction channel-permeable dye and expression of Cx43 in the prefrontal cortex in rats subjected to chronic unpredictable stress. Secondly, on the other side treatment with antidepressants also results in changes in the expression level of Cx43 in astrocytes as indicated in **Table 1**. Five antidepressants, from three different therapeutic classes, have been tested in cellular and animal models; results indicate that 24–48 h treatment induces an increase in Cx43 expression at mRNA and/or protein levels. This is particularly the case for fluoxetine that has been tested in several models (Fatemi et al., 2008; Mostafavi et al., 2008; Sun et al., 2012). However, there is little information about the effect of these drugs on the functional status of gap junctional communication and none about hemichannel activity. So far based on this literature it is tempting to deduce and summarize that depressive brains show a down-regulation of Cx43 while antidepressant treatments favor its up-regulation (see Rajkowska and Stockmeier, 2013). Nevertheless, an important clue concerning Cxs is to identify the functional consequences of these treatments on Cx43-based channels since changes in level of expression can have unpredictable consequences on their function. In order to address this question we have carried out a systematic test of seven antidepressants on gap junctional communication and hemichannel activity in primary cultures of astrocytes, known to express only Cx43 (Dermietzel et al., 1991; Giaume et al., 1991; Koulakoff et al., 2008). The present study indicates that when used at a non-toxic and clinically relevant concentration they have differential effects on both channel functions leading to a more complicated global view of their action on intercellular communication mediated by Cx43 in astrocytes.

**Table 1:** Summary table of the effects of antidepressants on Cx43 expression and gap junctional function in astrocytes.

Antidepressant	Class	Effect on Cx43 expression	Effect on Cx43 function	Model	Treatment (dose and time)	Reference
Amitriptyline	TCA	Increase (mRNA, protein)	Increase (gap junction)	Primary cultures rat cortical astrocytes	25 μM, 48 h	Morioka et al., 2014


Clomipramine	TCA	Increase (protein)	NT	Primary culture rat cortical astrocytes	10 μM, 48 h	Morioka et al., 2014


Fluoxetine	SSRI	Increase (protein)	NT	Rat *in vivo* Prefrontal cortex	20 mg/kg i.p. for 21 days	Fatemi et al., 2008


Fluoxetine	SSRI	Increase (mRNA, protein)	NT	Human astrocytoma cell line	10, 20 μg/ml 24 h	Mostafavi et al., 2008


Fluoxetine	SSRI	Increase (protein)	No effect (gap junction)	Rat *in vivo* Prefrontal cortex	10 mg/kg 21 days	Sun et al., 2012


Duloxetine	SNRI	Increase (mRNA, protein)	No effect (gap junction)	Rat *in vivo* Prefrontal cortex	10 mg/kg 21 days	Sun et al., 2012


Fluvoxamine	SSRI	Increase (protein)	NT	Primary cultures rat cortical astrocytes	25 μM 48 h	Morioka et al., 2014

## Materials and Methods

All experiments were performed according to the European Community Council Directives of 2010/63/UE and all efforts were made to minimize the number of animals. This study was carried out in accordance with the recommendations of the Ethic Committee 59, Paris, France and received the approval of the Scientific Committee of the animal facilities of the Collège de France.

### Cortical Astrocyte Cultures

Primary astrocyte cultures were prepared from the cortex of newborn (1–2 days) OF1 mice as previously described (Meme et al., 2006). For western blot and scrape-loading dye-transfer experiments, cells were seeded on polyornithine-coated 35-mm-diameter dishes (Nunc, Roskilde, Denmark) at a density of 5 × 10^5^cells/mL. For hemichannel experiments cells were seeded (2 × 10^5^ cells per well) on glass coverslips (Gassalem, Limeil-Brévannes, France) placed inside 24-round-well plate; area 1.9 cm2/well; (NunClon, Thermoscientific, Atlanta, GA, USA). Cellular medium, DMEM (Sigma–Aldrich, St-Louis MO, USA), supplemented with penicillin (5 U/ml), streptomycin (5 μg/ml; Invitrogen, Carlsbad, CA, USA), fungizone amphotericin B (500 ng/mL; Gibco, Life Technologies, Carlsbad, CA, USA), and 10% FCS (Hyclone, Logan, UT, USA), was changed twice a week. When cells reached confluence, around 10 days *in vitro* (DIV), they were harvested with trypsin-EDTA (Invitrogen). The medium was changed twice a week until the experiments were carried out. In order to characterize the proportion of microglia in primary culture of astrocytes, the two cell types were identified by immunostaining with Isolectine B4 and GFAP antibodies, respectively.

### Products and Cell Treatments

Astrocyte cultures were treated for 24 h with lipopolysaccharide (LPS, 1 μg/ml) and/or the following antidepressants: fluoxetine, duloxetine, paroxetine, reboxetine, amitriptyline, imipramine, venlafaxine (5, 10, or 20 μM, Sigma–Aldrich, Saint-Louis, MO, USA). Carbenoxolone (50 μM, Sigma–Aldrich) was used as positive inhibitor control for gap junction channels. Drugs were prepared either in H_2_0 or DMSO. Control cells received no treatment and were previously studied with vehicle (H20 or DMSO) which induced no changes in comparison with untreated cells.

### Determination of Gap Junctional Communication

Experiments were performed by using the scrape-loading dye-transfer technique, as previously described (Meme et al., 2006). Briefly, cells were incubated at room temperature for 10 min in HEPES buffered salt solution containing (in mM): NaCl, 140; KCl, 5.4; CaCl_2_, 1.8; MgCl_2_, 1; glucose, 10; HEPES, 5 at pH 7.4. Cells were then washed with a calcium-free HEPES solution for 1 min and the scrape loading and dye transfer assay (see Giaume et al., 2012) was carried out in the same calcium-free solution containing Lucifer yellow CH (427 Da, 1 mg/ml). One minute after scraping procedure, cells were washed with the HEPES solution and then Lucifer yellow loaded in the cells was allowed to diffuse through gap junction channels for 8 min. Photomicrographs were taken and data were quantified using NIS Nikon software. In all experiments, the fluorescence area of the first row of cells initially loaded, as measured in the presence of the gap junction channel inhibitor carbenoxolone (50 μM, 24 h), was subtracted from the total fluorescence area.

### Ethidium Bromide Uptake Experiments in Cortical Astrocyte Cultures

Following 10 min exposure to 5 μM ethydium bromide (EtBr), cells were washed with HEPES buffered salt solution containing (in mM): NaCl, 140; KCl, 5.4; CaCl_2_, 1.8; MgCl_2_, 1; glucose, 10; HEPES, 5 at pH 7.4. After 10 min in fixing solution (4% paraformaldehyde in 0.12 M buffer phosphate) and rinsing with phosphate buffered saline (PBS), cells were mounted in Fluoromount-G mounting medium (Orellana et al., 2011). Images of astrocyte cultures were taken with a 40× objective using a confocal laser-scanning microscope (Leica TBCS SP5). Stacks of consecutive confocal images for 10 μm at 0.49 μm intervals were acquired with an argon ion laser at 488 nm. Confocal images of EthBr uptake were analyzed with Image J software. The EtBr fluorescence intensity in the nuclei of astrocytes in each image was measured and the average of six images of different areas in the same culture was calculated the final measurement of dye uptake in that culture.

### Western Blot

After 24 h of treatment, cultures were rinsed with PBS 1X and added 75 μL of a solution containing protease and phosphatase inhibitors (orthovanadate 1 mM; α-glycerophosphate 10 mM), and complete miniprotease inhibitor (Roche Diagnostics, Meylan, France). Cells were then harvested by scraping with a rubber policeman and pelleted cells were added 20 μL of 5X Laemmli sample buffer. Samples were boiled for 5 min, placed on ice, and lysed by sonication (Ultrasonic cell disrupter, Microson, Bruxelles, Belgium). Then, samples were stored at -20°C. Proteins were measured with the Bio-Rad protein assay (Bio-Rad laboratories, Richmond, CA, USA). For each cell lysate sample, 20 μg of proteins were separated on Bis-Tris 4–12% NuPAGE gels and electro-transferred to nitrocellulose sheets as previously described (Orellana et al., 2011). Non-specific protein binding was blocked by incubation of nitrocellulose sheets in tris-buffered saline (TBS) – Tween – milk solution (500 mL TBS 1X; 500 μL Tween 20X; non-fat powder milk 25 g) for 1 h. Blots were then incubated overnight with primary antibody mouse Cx43 1:500 (Transduction Laboratories, Le Pont de Claix, France) at 4°C, followed by 4 × 15 min PBS washes. Blots were incubated with goat anti-mouse antibody 1:2500 conjugated to horseradish peroxidase (Tébu, Le Perray-En-Yveline, France). Immunoreactivity was detected by ECL detection using the SuperSignal kit (Pierce, Rockford, IL, USA) according to instructions. Blots were then reprobed with mouse monoclonal anti-glyceraldehyde 3-phosphate dehydrogenase peroxidase (Sigma–Aldrich, 1:10,000) to check the protein load. Chemiluminescence imaging was performed on a LAS4000 (Fujifilm, Stamford, CT, USA). Semiquantitative densitometric analysis was performed with ImageJ software after scanning the bands.

### Statistical Analysis

For each data group, results are expressed as mean ± SEM and *n* refers to the number of independent experiments. Kruskal–Wallis test and one-way ANOVA, followed, respectively, by Dunn and Bonferroni post tests, were used as well as unpaired *t*-test. Differences are considered significant at ^∗^*P* < 0.05, ^∗∗^*P* < 0.01, ^∗∗∗^*P* < 0.001 versus control, +*P* < 0.05, ++*P* < 0.01, and +++*P* < 0.001 vs. LPS. GraphPad Prism 5 software (GraphPad Software, La Jolla, CA, USA) was used for calculations.

## Results

**FIGURE 1:**
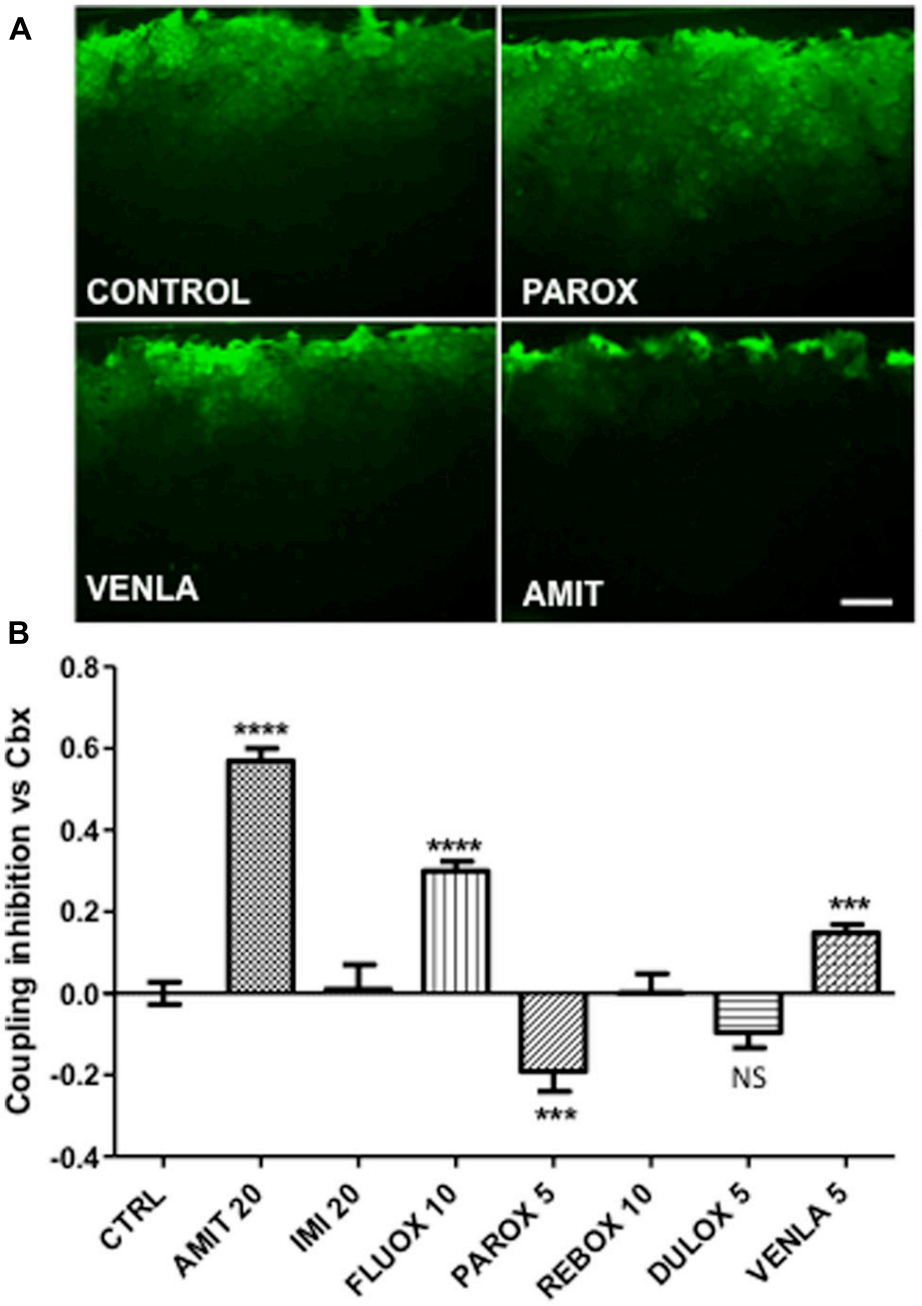
**Modulation of astrocyte Cx43 gap junctional communication after 24-h treatment by seven antidepressants in mice cortical astrocyte cultures**. Amitriptyline (AMIT), imipramine (IMI), fluoxetine (FLUOX), paroxetine (PAROX), reboxetine (REBOX), duloxetine (DULOX), and venlafaxine (VENLA) were added from 5 to 20 μM in cellular medium during 24 h, gap junctional communication was evaluated by the scrape loading dye transfer method. **(A)** Pictures of control, paroxetine 5 μM, venlafaxine 5 μM, and amitriptyline 20 μM, illustrate the Lucifer yellow spreading through astrocyte gap junctions for these different treatments. **(B)** Summary diagram of junctional communication in vehicle-treated cells (control group) and after antidepressant treatment. Note that the values are normalized to carbenoxolone (50 μM, 24 h). Data are the means ± SEM *n* = 3–7 per group, ^∗∗∗^*p* < 0.001, ^∗∗∗∗^*p* < 0.0001 vs. control, Kruskal–Wallis test and one-way ANOVA followed, respectively, by Dunn and Bonferroni post test. Scale bar 20 μM.

The doses of the antidepressants were chosen in accordance with literature addressing the neuropharmacokinetics of the tested molecules of interest. Accordingly, cultured cortical astrocytes were treated with concentrations identical to those reported for brains of human or rodent after treatment with clinically relevant doses from *in vivo* studies: for fluoxetine, 20 μM in human brain is achieved at 20 mg/day (see Henry et al., 2005); for venlafaxine, 10 μM in mice brain is reached at 20 mg/kg (Karlsson et al., 2011); for duloxetine, 4.2 mg/kg in rat leads to 10 μM in brain (Kielbasa and Stratford, 2012). For the other molecules (amitriptyline, imipramine, paroxetine, reboxetine) the doses were selected below cell toxicity that was identified by microscopic examination of astrocyte cultures treated for 24 h, related to changed cell morphology and entry of Lucifer yellow into damaged cells. More precisely the lack of toxicity of the selected doses was routinely validated by the absence of Lucifer yellow unspecific uptake in area far from the scrape lines in scrape-loading dye-transfer experiments (see **Figure 1A**). Based on these criterions all molecules were tested at concentrations between 5 and 20 μM (24 h) for toxicity; we observed that fluoxetine at 20 μM, paroxetine 10 μM, and duloxetine 20 μM were toxic at these indicated doses, consequently these molecules were tested at lower doses.

The first question addressed during this screening with antidepressants was their effect on the level of Cx43 expression investigated by western blotting. As indicated in **Table 2**, the seven tested antidepressants did not significantly modify the level of expression of Cx43 studied in cultured cortical mouse astrocytes (*n* = 3–6 per group, *p* > 0.05 One way ANOVA, Dunn post test).

**Table 2:** Summary table of the effect of seven antidepressants for four different classes: tricyclic (TCA), selective serotonin reuptake inhibitor (SSRI), noradrenaline reuptake inhibitor (NRI), serotonin noradrenaline reuptake inhibitor (SNRI), on Cx43 expression and channel functions in cultured astrocytes.

Antidepressant	Class	Concentration tested (μM)	Cx43 expression	Effect on gap junctional coupling	Effect on hemichannels
Amitriptyline	TCA	20	No significant effect	Inhibition	Mild inhibition
Imipramine	TCA	20	No significant effect	No effect	Mild inhibition
Fluoxetine	SSRI	10	No significant effect	Inhibition	Total inhibition
Paroxetine	SSRI	5	No significant effect	Increase	Total inhibition
Reboxetine	NRI	10	No significant effect	No effect	Mild inhibition
Duloxetine	SNRI	5	No significant effect	No effect	Total inhibition
Venlafaxine	SNRI	5	No significant effect	Inhibition	Mild inhibition	

The effect of the selected antidepressants on gap junctional communication was then tested by using the scrape-loading dye transfer technique (see Giaume et al., 2012). In control condition after 10 min the Lucifer yellow diffuses widely perpendicular to the scrape line that indicates a high level of gap junctional communication (**Figure 1A**, control). As illustrated in **Figure 1B**, three different effects were observed. Three antidepressants, amitriptyline (20 μM; **Figure 1A**), fluoxetine (10 μM), and venlafaxine (5 μM; **Figure 1A**) reduced intercellular dye spread by 57% (*n* = 6), 25% (*n* = 6), and 15% (*n* = 6), respectively. In contrast, paroxetine (5 μM; **Figure 1A**) increased dye coupling by 19% (*n* = 7). Finally, imipramine (20 μM, *n* = 3), reboxetine (10 μM, *n* = 6) and duloxetine (5 μM, *n* = 9) had no statistically significant action on the level of intercellular communication between astrocytes, neither at these doses nor at higher non-toxic doses.

**FIGURE 2:**
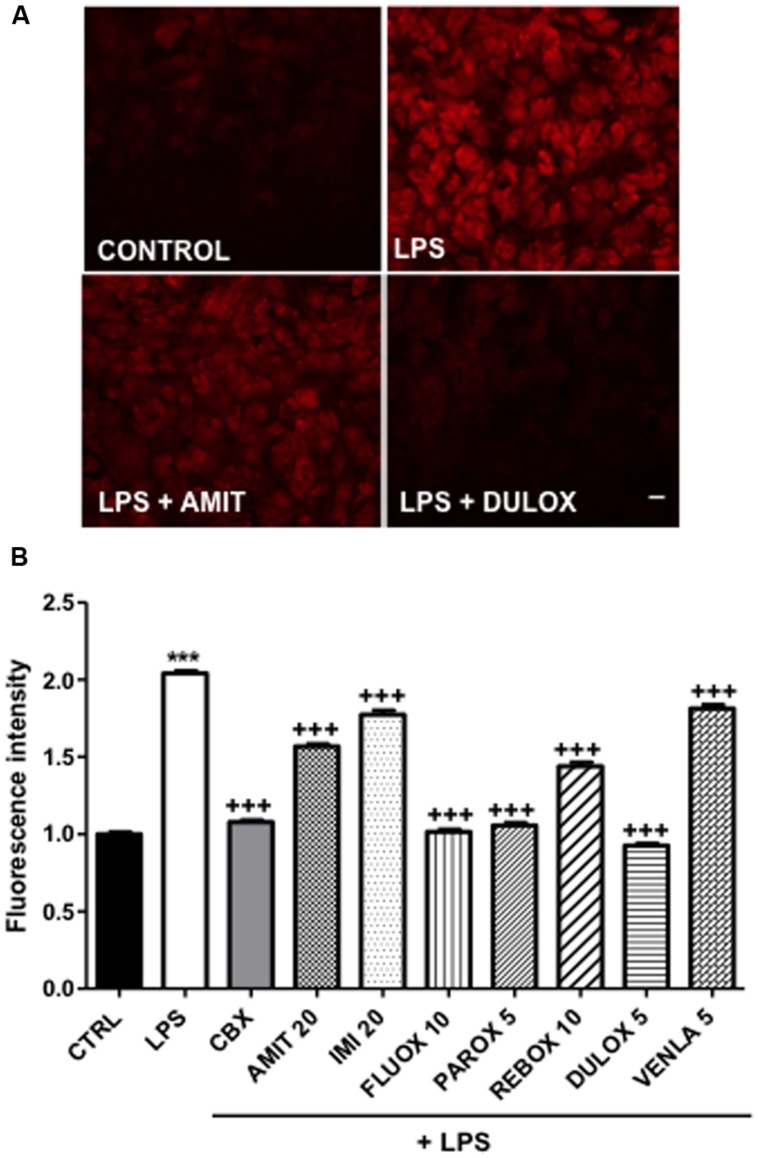
**Regulation of hemichannel activity after 24-h antidepressant treatments in mice cortical astrocytic cultures**. LPS 1 μg/mL was added 24-h before experiment to induce hemichannel opening and hemichannel activity was evaluated by using the ethidium bromide uptake assay. **(A)** Pictures of control, LPS alone, LPS + amitriptyline 20 μM, and LPS + duloxetine 5 μM uptake experiments. **(B)** Summary diagram of hemichannel activity in untreated astrocytes (CTRL) and after treatments with amitriptyline (AMIT), imipramine (IMI), fluoxetine (FLUOX), paroxetine (PAROX), reboxetine (REBO), duloxetine (DULOX), and venlafaxine (VENLA) were also added from 5 to 20 μM in cellular medium during 24 h. Carbenoxolone (CBX, 50 μM, 24 h) was used as a positive control for the inhibition of Cx43 hemichannels. Data are the means ± SEM *n* = 3–6 per group, ^∗∗∗^*p* < 0.001 vs. control, +++*p* < 0.001 vs. LPS, One-way ANOVA followed by Dunn post test. Scale bar 20 μM.

In normal condition, astrocytes in culture as well as in acute slices are characterized by a high level of gap junctional communication and low hemichannel activity (Bennett et al., 2003; Retamal et al., 2007; but see Chever et al., 2014). However, in most pathological situations involving brain inflammation, a reactive gliosis is associated with elevated hemichannel activity in astrocytes (see Bennett et al., 2012; Giaume et al., 2013). Such low hemichannel activity was also observed in our culture condition (**Figure 2A**). Therefore, to induce hemichannel activity we treated our cortical primary cultures, in which 11% (*n* = 9) of isolectin B4-positive microglia versus GFAP-positive astrocytes were present, with the endotoxin LPS. As already reported for *in vitro* astrocytes (Retamal et al., 2007), we observed that LPS treatment (1 μg/ml, 24 h) inhibited gap junctional communication by 64% (*n* = 6), in such condition we found that the antidepressants amitriptyline (20 μM, *n* = 4), imipramine (20 μM, *n* = 4), venlafaxine (5 μM, *n* = 3), and duloxetine (5 μM, *n* = 4) did not reverse the inhibition induced by LPS, when co-treated during 24 h in astrocyte cultures. However, paroxetine (5 μM, *n* = 4) and reboxetine (10 μM, *n* = 3) reversed LPS-induced uncoupling, in a low but significant manner, respectively, by 17% (*n* = 4) and 10% (*n* = 3) whereas fluoxetine (10 μM) improved it by 10% (*n* = 4; data not illustrated). Moreover, we found that LPS treatment (1 μg/ml, 24 h) increased by 104% (*n* = 7) the uptake of ethidium bromide (EtBr) in GFAP-positive astrocytes indicating that, as previously reported (Retamal et al., 2007; Abudara et al., 2015) hemichannels in cortical astrocytes were activated (**Figure 2A**). As expected EtBr uptake was inhibited by 93% (*n* = 3) in the presence of carbenoxolone (50 μM; 24 h) indicating that this uptake is mediated through hemichannel activity (**Figure 2B**). All the seven antidepressants tested had a significant inhibitor effect on the LPS-induced EtBr uptake, however, with difference in their efficiency. Indeed, fluoxetine (10 μM), paroxetine (5 μM), and duloxetine (5 μM; **Figure 2A**) had pronounced effect and inhibited EtBr uptake by 97% (*n* = 3), 93% (*n* = 3), and 111% (*n* = 4), respectively (**Figure 2B**). Meanwhile, amitriptyline (20 μM; **Figure 2A**), imipramine (20 μM), reboxetine (10 μM), and venlafaxine (5 μM) had milder effect with 45% (*n* = 6), 28% (*n* = 3), 52% (*n* = 3), and 23% (*n* = 4), respectively (**Figure 2B**).

## Discussion

The objective of the present study was to investigate the effects of a panel of antidepressants on the property of the astroglial Cx43, a membrane protein widely expressed in glial cells that contributes actively to neuroglial interaction in normal and disease brain (see Ransom and Giaume, 2013). For this purpose we undertook an *in vitro* screening of seven antidepressants from four different classes (TCA, SSRI, NRI, SNRI), all implied in serotonin and/or noradrenaline reuptake inhibition, to characterize their action on astroglial Cx43 at diverse levels: protein expression, gap junctional communication and hemichannel function. We selected a simple cellular system, primary cultures of mouse cortical and striatal astrocytes in which only Cx43 is detected (Dermietzel et al., 1991; Giaume et al., 1991; Koulakoff et al., 2008). The tested concentrations were determined either based on the literature, when clinically relevant doses were available, or by establishing experimentally their threshold of toxicity for 24 h treatment (see Results section). While we did not observe any significant change in Cx43 protein level, we found that antidepressants had differential effects on astroglial Cx43-based gap junctional communication as summarized in **Table 2**. These results are different compared those reported in the few articles that so far have addressed these properties for some of the antidepressants used in the present study, i.e., amitriptyline, fluoxetine, and duloxetine (see **Tables 1** and **2**). Indeed, Sun et al. (2012) and Morioka et al. (2014) reported, respectively, an enhancement in Cx43 mRNA and protein levels in astrocytes from cultured rat astrocytes treated with amitriptyline and in the prefrontal cortex of rats chronically treated with duloxetine (Sun et al., 2012; Morioka et al., 2014). Additionally, two studies have also shown a rise in Cx43 protein levels in the prefrontal cortex after *in vivo* treatment with fluoxetine (Fatemi et al., 2008; Sun et al., 2012), as well as in human astrocytoma cultures (Mostafavi et al., 2008). However, no changes in Cx43 gap junctional communication have been found after *in vivo* treatments with both fluoxetine and duloxetine in rats (Sun et al., 2012) whereas this Cx43 function was increased after amitriptyline treatment in cultured rat astrocytes (Morioka et al., 2014). In our experiments (see **Table 2**) only paroxetine (5 mM) was found to increase gap junctional communication while duloxetine (5 μM) has a slight, but not statistically significant increasing effect. Imipramine (20 μM) and reboxetine (10 μM) had no effect and an inhibition was observed with amitriptyline (20 μM), fluoxetine (10 μM), and venlafaxine (5 μM). These effects were not specific to a defined class of antidepressants and allow for taking a step back regarding results from other models. Indeed, our study demonstrates that the link between antidepressants and Cx43-mediated intercellular communication in astrocyte is likely more complex than the literature consensual interpretation concerning an increase of Cx43 expression and function, and that opposed effects are observed within a same therapeutic class. However, when pointing out the differences between our results and the current literature, several parameters must be taken into account. First, the models of study differed: we used cultured mouse astrocytes, while Sun et al. (2012) and Fatemi et al. (2008) worked *in vivo* on rats, and others used cell culture models from various species and cellular types in culture (human astrocytoma and rat astrocytes). Second, dosage, time, and chronicity of the treatments also differ. Indeed, our *in vitro* tested concentrations were non-toxic and similar to what is found in the brain in pharmacokinetics studies addressing clinically relevant doses for these molecules, and ranged from 1 to 100 μM in the literature. In literature, *in vitro* treatments were administered for several minutes to 48 h (see **Table 1**). We treated for 24 h, as for example preliminary trials of 48-h treatments with amitriptyline (20 μM) induced cell toxicity. In addition, in the studies that were performed using *in vivo* models, animals received chronic treatment (see **Table 1**) that certainly involved more integrated and complex mechanisms. Taken as a whole these information complete and extend the knowledge about the effect of antidepressants on gap junctional communication in astrocytes. Finally, as no changes in Cx43 expression were detected after treatment with antidepressant, we suggested that they act at the post-translational level.

Up-to-now there was no indication about the effect of antidepressants on the other channel function of Cx43 in astrocytes, i.e., the hemichannel activity. This lack of information can be attributed to the fact that for astrocytes this activity has been established much latter than the gap junction channel function and that hemichannels are weakly opened in normal conditions hence requiring a pathological context to be activated (see Bennett et al., 2003; Giaume et al., 2013). We confirmed this feature on untreated primary cortical cultures as the Cx channel inhibitor carbenoxolone had no significant no effect (data not shown) on the uptake of EtBr (see Giaume et al., 2012). However, we took advantage that our LPS-stimulated cultures contained a non-negligible proportion of microglial cells (11%), a situation already identified to activated Cx43 hemichannels and to reduce gap junctional communication in astrocytes (Retamal et al., 2007). Indeed, we previously showed that when LPS-stimulated microglia are co-cultured with astrocytes two pro-inflammatory cytokines are released, i.e., TNF-α and IL-1β, activating Cx43 hemichannel activity in astrocytes (Retamal et al., 2007; Abudara et al., 2015). Interestingly, LPS has been reported to cause time-dependent behavioral alterations with sickness behavior (Huang et al., 2008) and a depressive-like behavior observed 24 h after LPS challenge (Painsipp et al., 2011; Custódio et al., 2013); and it is noteworthy that this effect is reversed by fluoxetine (Yirmiya et al., 2001), imipramine (Tomaz et al., 2014), paroxetine and duloxetine (Ohgi et al., 2013). Besides, the level of TNF-α and IL-1β, are increased after LPS treatments in animal model, similarly to the increase observed in subjects with major depression disease (Seidel et al., 1995; Sluzewska et al., 1996; Dowlati et al., 2010; Hannestad et al., 2011). On this basis LPS has been proposed as an inducer of depressive-like context (Mello et al., 2013; Ohgi et al., 2013) confirming the interest of LPS in the evaluation of antidepressant mechanisms. Using the EtBr uptake assay we found that all tested antidepressants had an inhibitory effect on LPS-induced astroglial hemichannel activity. The effect on Cx43-based hemichannel function was correlated when considering the class of the antidepressants, the SSRIs (fluoxetine, paroxetine) induced a strong inhibition while the TCAs (amitriptyline, imipramine) and NRI (reboxetine) had mild effect, the treatment with SNRI (duloxetine, venlafaxine) resulting in mixed inhibition efficiency. Interestingly, several of these antidepressants are known to have an inhibitory effect on the production of pro-inflammatory cytokines, in particular TNF-α and IL-1β, fluoxetine (Chiou et al., 2006; Tai et al., 2009; Valera et al., 2014), amitriptyline (Obuchowicz et al., 2006; Tai et al., 2009), paroxetine (Liu et al., 2014), and imipramine (Lee et al., 2012). Meanwhile venlafaxine, which presents the lowest inhibitory effect on LPS-induced hemichannel activity (23%), is the only tested compound that increased the level of TNF-α (Valera et al., 2014). Taken as a whole these results could support the idea that antidepressants may control Cx43 hemichannel activity through the production of TNF-a and/or IL-1β as the result of a reduction of microglial activation. However, just paroxetine and reboxetine modestly reversed the inhibition of gap junctional communication induced by LPS, it could consequently imply that antidepressant effects are not targeting microglia-released interleukins. It could also suggest that the antidepressant inhibitory effects on hemichannels are acting downstream to the microglial step and/or more directly on the Cx43 hemichannel function. Nevertheless, more work is needed to decipher the specific mechanisms involved in the regulation of astroglial Cx channels by antidepressants, an aim that is beyond the scope of the present study.

Interestingly, hemichannel activity in astrocytes has been shown to provide a pathway for glutamate release (Ye et al., 2003; Abudara et al., 2015). Consequently, the antidepressant inhibitory action on hemichannel activity could support the current hypothesis of the action of these drugs on glutamine/glutamate metabolic cycle (Garakani et al., 2013) and glutamate transmission (Gorman and Docherty, 2010; Sanacora and Banasr, 2013) in the pathophysiology of major depression (Etiévant et al., 2013). Finally, glutamate gliotransmission mediated by Cx43 hemichannels has been recently reported to occur in a model of chronic restraint stress (Orellana et al., 2015) which is admitted as a model inducing depressive-like symptoms in rodents (Levinstein and Samuels, 2014).

The present study reflects the need to re-evaluate the statement according to which an alternative strategy for antidepressive treatments is to target astroglial Cx43 and to increase gap junctional communication. This proposition was solely based on reports indicating that several antidepressants favor Cx43 expression levels while a few have investigated their effect on the functional aspect, i.e., gap junctional communication. Based on the present results the effect of antidepressant drugs on astroglial gap junctions appears more complex than initially thought and suggests that Cx43 hemichannel activity in astrocytes may be part of the mode of action of these drugs. Finally, our observation, and in particular those related to hemichannel activity, could benefit to the understanding of the mode of action of antidepressants in other pathologies treated by antidepressants such as neuropathic pain (Dworkin et al., 2010; Finnerup et al., 2015) and for which the involvement of glial Cx43 hemichannel activity has been proposed (Chen et al., 2014).

## Author Contributions

TJ and CG contributed to the study design, TJ and AP conducted the experiments, CG wrote the initial draft; TJ, CG, FM, MC contributed to the writing of the manuscript.

## Conflict of Interest Statement

The authors declare that the research was conducted in the absence of any commercial or financial relationships that could be construed as a potential conflict of interest.

## References

[B1] Abudara V. Roux L. Dallérac G. Matias I. Dulong J. Mothet J. P. (2015). Activated microglia impairs neuroglial interaction by opening Cx43 hemichannels in hippocampal astrocytes. *Glia* 63 795–811. 10.1002/glia.22785 25643695

[B2] Araque A. Parpura V. Sanzgiri R. P. Haydon P. G. (1999). Tripartite synapses: glia, the unacknowledged partner. *Trends Neurosci.* 22 208–215. 10.1016/S0166-2236(98)01349-6 10322493

[B3] Bennett M. V. Contreras J. E. Bukauskas F. F. Sáez J. C. (2003). New roles for astrocytes: gap junction hemichannels have something to communicate. *Trends Neurosci.* 26 610–617. 10.1016/j.tins.2003.09.008 14585601 PMC3694339

[B4] Bennett M. V. Garré J. M. Orellana J. A. Bukauskas F. F. Nedergaard M. Giaume C. (2012). Connexin and pannexin hemichannels in inflammatory responses of glia and neurons. *Brain Res.* 1487 3–15. 10.1016/j.brainres.2012.08.042 22975435 PMC3627726

[B5] Bernard R. Kerman I. A. Thompson R. C. Jones E. G. Bunney W. E. Barchas J. D. (2011). Altered expression of glutamate signaling, growth factor, and glia genes in the locus coeruleus of patients with major depression. *Mol. Psychiatry* 16 634–646. 10.1038/mp.2010.44 20386568 PMC2927798

[B6] Chen G. Park C. K. Xie R. G. Berta T. Nedergaard M. Ji R. R. (2014). Connexin-43 induces chemokine release from spinal cord astrocytes to maintain late-phase neuropathic pain in mice. *Brain* 137(Pt 8), 2193–2209. 10.1093/brain/awu140 24919967 PMC4107738

[B7] Chever O. Lee C. Y. Rouach N. (2014). Astroglial connexin43 hemichannels tune basal excitatory synaptic transmission. *J. Neurosci.* 34 11228–11232. 10.1523/JNEUROSCI.0015-14.2014 25143604 PMC6615508

[B8] Chiou S. H. Chen S. J. Peng C. H. Chang Y. L. Ku H. H. Hsu W. M. (2006). Fluoxetine up-regulates expression of cellular FLICE-inhibitory protein and inhibits LPS-induced apoptosis in hippocampus-derived neural stem cell. *Biochem. Biophys. Res. Commun.* 343 391–400. 10.1016/j.bbrc.2006.02.180 16545775

[B9] Colangelo A. M. Alberghina L. Papa M. (2014). Astrogliosis as a therapeutic target for neurodegenerative diseases. *Neurosci. Lett.* 565 59–64. 10.1016/j.neulet.2014.01.014 24457173

[B10] Cotrina M. L. Nedergaard M. (2012). Brain connexins in demyelinating diseases: therapeutic potential of glial targets. *Brain Res.* 1487 61–68. 10.1016/j.brainres.2012.07.003 22789906 PMC3506183

[B11] Custódio C. S. Mello B. S. Cordeiro R. C. de Araújo F. Y. Chaves J. H. Vasconcelos S. M. (2013). Time course of the effects of lipopolysaccharide on prepulse inhibition and brain nitrite content in mice. *Eur. J. Pharmacol.* 713 31–38. 10.1016/j.ejphar.2013.04.040 23665499

[B12] Dermietzel R. Hertberg E. L. Kessler J. A. Spray D. C. (1991). Gap junctions between cultured astrocytes: immunocytochemical, molecular, and electrophysiological analysis. *J. Neurosci.* 11 1421–1432. 1851221 10.1523/JNEUROSCI.11-05-01421.1991 PMC6575331

[B13] Dowlati Y. Herrmann N. Swardfager W. Liu H. Sham L. Reim E. K. (2010). A meta-analysis of cytokines in major depression. *Biol. Psychiatry* 67 446–457. 10.1016/j.biopsych.2009.09.033 20015486

[B14] Dworkin R. H. O’Connor A. B. Audette J. Baron R. Gourlay G. K. Haanpää M. L. (2010). Recommendations for the pharmacological management of neuropathic pain: an overview and literature update. *Mayo Clin. Proc.* 85 S3–S14. 10.4065/mcp.2009.0649 20194146 PMC2844007

[B15] Ernst C. Nagy C. Kim S. Yang J. P. Deng X. Hellstrom I. C. (2011). Dysfunction of astrocyte connexins 30 and 43 in dorsal lateral prefrontal cortex of suicide completers. *Biol. Psychiatry* 70 312–319. 10.1016/j.biopsych.2011.03.038 21571253

[B16] Etiévant A. Lambás-Señas L. Scarna H. Lucas G. Haddjeri N. (2013). Astrocytes and gliotransmitters: new players in the treatment of major depression? *Curr. Drug Targets* 14 1295–1307. 24010966 10.2174/13894501113149990197

[B17] Fatemi S. H. Folsom T. D. Reutiman T. J. Lee S. (2008). Expression of astrocytic markers aquaporin 4 and connexin 43 is altered in brains of subjects with autism. *Synapse* 62 501–507. 10.1002/syn.20519 18435417 PMC2697054

[B18] Finnerup N. B. Attal N. Haroutounian S. McNicol E. Baron R. Dworkin R. H. (2015). Pharmacotherapy for neuropathic pain in adults: a systematic review and meta-analysis. *Lancet Neurol.* 14 162–173. 10.1016/S1474-4422(14)70251-0 25575710 PMC4493167

[B19] Freitas-Andrade M. Naus C. C. (2015). Astrocytes in neuroprotection and neurodegeneration: the role of connexin43 and pannexin1. *Neuroscience* 10.1016/j.neuroscience.2015.04.035 [Epub ahead of print]. 25913636

[B20] Froger N. Orellan J. A. Calvo C. F. Amigou E. Kozoriz M. G. Naus C. C. (2010). Inhibition of cytokine-induced connexin43 hemichannel activity in astrocytes is neuroprotective. *Mol. Cell. Neurosci.* 45 37–46. 10.1016/j.mcn.2010.05.007 20684043

[B21] Garakani A. Martinez J. M. Yehuda R. Gorman J. M. (2013). Cerebrospinal fluid levels of glutamate and corticotropin releasing hormone in major depression before and after treatment. *J. Affect. Disord.* 146 262–265. 10.1016/j.jad.2012.06.037 22840611

[B22] Giaume C. Fromaget C. el Aoumari A. Cordier J. Glowinski J. Gros D. (1991). Gap junctions in cultured astrocytes: single-channel currents and characterization of channel-forming protein. *Neuron* 6 133–143. 10.1016/0896-6273(91)90128-M 1702648

[B23] Giaume C. Kirchhoff F. Matute C. Reichenbach A. Verkhratsky A. (2007). Glia: the fulcrum of brain diseases. *Cell Death Differ.* 14 1324–1335. 10.1038/sj.cdd.4402144 17431421

[B24] Giaume C. Leybaert L. Naus C. C. Sáez J. C. (2013). Connexin and pannexin hemichannels in brain glial cells: properties, pharmacology, and roles. *Front. Pharmacol.* 4:88 10.3389/fphar.2013.00088 PMC3713369 23882216

[B25] Giaume C. Orellana J. A. Abudara V. Sáez J. C. (2012). Connexin-based channels in astrocytes: how to study their properties. *Methods Mol. Biol.* 814 283–303. 10.1007/978-1-61779-452-0_19 22144314

[B26] Gorman J. M. Docherty J. P. (2010). A hypothesized role for dendritic remodeling in the etiology of mood and anxiety disorders. *J. Neuropsychiatry Clin. Neurosci.* 22 256–264. 10.1176/appi.neuropsych.22.3.256 20686132

[B27] Halassa M. M. Haydon P. G. (2010). Integrated brain circuits: astrocytic networks modulate neuronal activity and behavior. *Annu. Rev. Physiol.* 72 335–355. 10.1146/annurev-physiol-021909-135843 20148679 PMC3117429

[B28] Hannestad J. DellaGioia N. Bloch M. (2011). The effect of antidepressant medication treatment on serum levels of inflammatory cytokines: a meta-analysis. *Neuropsychopharmacology* 36 2452–2459. 10.1038/npp.2011.132 21796103 PMC3194072

[B29] Harris A. L. (2007). Connexin channel permeability to cytoplasmic molecules. *Prog. Biophys. Mol. Biol.* 94 120–143. 10.1016/j.pbiomolbio.2007.03.011 17470375 PMC1995164

[B30] Henry M. E. Schmidt M. E. Hennen J. Villafuerte R. A. Butman M. L. Tran P. (2005). A comparison of brain and serum pharmacokinetics of R-fluoxetine and racemic fluoxetine: a 19-F MRS study. *Neuropsychopharmacology* 30 1576–1583. 10.1038/sj.npp.1300749 15886723

[B31] Huang Y. Henry C. J. Dantzer R. Johnson R. W. Godbout J. P. (2008). Exaggerated sickness behavior and brain proinflammatory cytokine expression in aged mice in response to intracerebroventricular lipopolysaccharide. *Neurobiol. Aging* 29 1744–1753. 10.1016/j.neurobiolaging.2007.04.012 17543422 PMC2647751

[B32] Kang J. Kang N. Lovatt D. Torres A. Zhao Z. Lin J. (2008). Connexin 43 hemichannels are permeable to ATP. *J. Neurosci.* 28 4702–4711. 10.1523/JNEUROSCI.5048-07.2008 18448647 PMC3638995

[B33] Karlsson L. Hiemke C. Carlsson B. Josefsson M. Ahlner J. Bengtsson F. (2011). Effects on enantiomeric drug disposition and open-field behavior after chronic treatment with venlafaxine in the P-glycoprotein knockout mice model. *Psychopharmacology (Berl.)* 215 367–377. 10.1007/s00213-010-2148-5 21191569

[B34] Kawasaki A. Hayashi T. Nakachi K. Trosko J. E. Sugihara K. Kotake Y. (2009). Modulation of connexin 43 in rotenone-induced model of Parkinson’s disease. *Neuroscience* 160 61–68. 10.1016/j.neuroscience.2009.01.080 19232380

[B35] Kielbasa W. Stratford R. E. Jr. (2012). Exploratory translational modeling approach in drug development to predict human brain pharmacokinetics and pharmacologically relevant clinical doses. *Drug Metab. Dispos.* 40 877–883. 10.1124/dmd.111.043554 22287668

[B36] Koulakoff A. Ezan P. Giaume C. (2008). Neurons control the expression of connexin 30 and connexin 43 in mouse cortical astrocytes. *Glia* 56 1299–1311. 10.1002/glia.20698 18512249

[B37] Koulakoff A. Mei X. Orellana J. A. Sáez J. C. Giaume C. (2012). Glial connexin expression and function in the context of Alzheimer’s disease. *Biochim. Biophys. Acta* 1818 2048–2057. 10.1016/j.bbamem.2011.10.001 22008509

[B38] Lee Y. H. Kim S. H. Kim Y. Lim Y. Ha K. Shin S. Y. (2012). Inhibitory effect of the antidepressant imipramine on NF-κB-dependent CXCL1 expression in TNFα-exposed astrocytes. *Int. Immunopharmacol.* 12 547–555. 10.1016/j.intimp.2012.01.011 22326584

[B39] Levinstein M. R. Samuels B. A. (2014). Mechanisms underlying the antidepressant response and treatment resistance. *Front. Behav. Neurosci.* 8:208 10.3389/fnbeh.2014.00208 PMC4073308 25018708

[B40] Liu R. P. Zou M. Wang J. Y. Zhu J. J. Lai J. M. Zhou L. L. (2014). Paroxetine ameliorates lipopolysaccharide-induced microglia activation via differential regulation of MAPK signaling. *J. Neuroinflammation* 11 47 10.1186/1742-2094-11-47 PMC3995780 24618100

[B41] Lundgaard I. Osório M. J. Kress B. T. Sanggaard S. Nedergaard M. (2014). White matter astrocytes in health and disease. *Neuroscience* 276 161–173. 10.1016/j.neuroscience.2013.10.050 24231735 PMC4016995

[B42] Mello B. S. Monte A. S. McIntyre R. S. Soczynska J. K. Custódio C. S. Cordeiro R. C. (2013). Effects of doxycycline on depressive-like behavior in mice after lipopolysaccharide (LPS) administration. *J. Psychiatr. Res.* 47 1521–1529. 10.1016/j.jpsychires.2013.06.008 23835040

[B43] Meme W. Calvo C. F. Froger N. Ezan P. Amigou E. Koulakoff A. (2006). Proinflammatory cytokines released from microglia inhibit gap junctions in astrocytes: potentiation by b -amyloid. *FASEB J.* 20 494–496. 16423877 10.1096/fj.05-4297fje

[B44] Miguel-Hidalgo J. J. Wilson B. A. Hussain S. Meshram A. Rajkowska G. Stockmeier C. A. (2014). Reduced connexin 43 immunolabeling in the orbitofrontal cortex in alcohol dependence and depression. *J. Psychiatr. Res.* 55 101–109. 10.1016/j.jpsychires.2014.04.007 24774648 PMC4078739

[B45] Morioka N. Suekama K. Zhang F. F. Kajitani N. Hisaoka-Nakashima K. Takebayashi M. (2014). Amitriptyline up-regulates connexin43-gap junction in rat cultured cortical astrocytes via activation of the p38 and c-Fos/AP-1 signalling pathway. *Br. J. Pharmacol.* 171 2854–2867. 10.1111/bph.12614 24641259 PMC4243860

[B46] Mostafavi H. Khaksarian M. Joghataei M. T. Hassanzadeh G. Soleimani M. Eftekhari S. (2008). Fluoxetin upregulates connexin 43 expression in astrocyte. *Basic Clin. Neurosci.* 5 74–79. 25436087 PMC4202606

[B47] Mylvaganam S. Ramani M. Krawczyk M. Carlen P. L. (2014). Roles of gap junctions, connexins, and pannexins in epilepsy. *Front. Physiol.* 5:172 10.3389/fphys.2014.00172 PMC4019879 24847276

[B48] Nagy J. I. Dudek F. E. Rash J. E. (2004). Update on connexins and gap junctions in neurons and glia in the mammalian nervous system. *Brain Res. Brain Res. Rev.* 47 191–215. 10.1016/j.brainresrev.2004.05.005 15572172

[B49] Naus C. C. Laird D. W. (2010). Implications and challenges of connexin connections to cancer. *Nat. Rev. Cancer* 10 435–441. 10.1038/nrc2841 20495577

[B50] Obuchowicz E. Kowalski J. Labuzek K. Krysiak R. Pendzich J. Herman Z. S. (2006). Amitriptyline and nortriptyline inhibit interleukin-1 release by rat mixed glial and microglial cell cultures. *Int. J. Neuropsychopharmacol.* 9 27–35. 10.1017/S146114570500547X 15963243

[B51] Ohgi Y. Futamura T. Kikuchi T. Hashimoto K. (2013). Effects of antidepressants on alternations in serum cytokines and depressive-like behavior in mice after lipopolysaccharide administration. *Pharmacol. Biochem. Behav.* 103 853–859. 10.1016/j.pbb.2012.12.003 23262300

[B52] Orellana J. A. Avendaño B. C. Montero T. D. (2014). Role of connexins and pannexins in ischemic stroke. *Curr. Med. Chem.* 21 2165–2182. 10.2174/0929867321666131228191714 24372216

[B53] Orellana J. A. Moraga-Amaro R. Díaz-Galarce R. Rojas S. Maturana C. J. Stehberg J. (2015). Restraint stress increases hemichannel activity in hippocampal glial cells and neurons. *Front. Cell. Neurosci.* 9:102 10.3389/fncel.2015.00102 PMC4382970 25883550

[B54] Orellana J. A. Shoji K. F. Abudara V. Ezan P. Amigou E. Sáez P. J. (2011). Amyloid β-induced death in neurons involves glial and neuronal hemichannels. *J. Neurosci.* 31 4962–4977. 10.1523/JNEUROSCI.6417-10.2011 21451035 PMC6622997

[B55] Painsipp E. Köfer M. J. Sinner F. Holzer P. (2011). Prolonged depression-like behavior caused by immune challenge: influence of mouse strain and social environment. *PLoS ONE* 6:e20719 10.1371/journal.pone.0020719 PMC3108969 21673960

[B56] Pannasch U. Vargová L. Reingruber J. Ezan P. Holcman D. Giaume C. (2011). Astroglial networks scale synaptic activity and plasticity. *Proc. Natl. Acad. Sci. U.S.A.* 108 8467–8472. 10.1073/pnas.1016650108 21536893 PMC3100942

[B57] Parpura V. Heneka M. T. Montana V. Oliet S. H. Schousboe A. Haydon P. G. (2012). Glial cells in (patho)physiology. *J. Neurochem.* 121 4–27. 10.1111/j.1471-4159.2012.07664.x 22251135 PMC3304021

[B58] Pérez-Alvarez A. Araque A. (2013). Astrocyte-neuron interaction at tripartite synapses. *Curr. Drug Targets* 14 1220–1224. 10.2174/13894501113149990203 23621508

[B59] Rajkowska G. Stockmeier C. A. (2013). Astrocyte pathology in major depressive disorder: insights from human postmortem brain tissue. *Curr. Drug Targets* 14 1225–1236. 10.2174/13894501113149990156 23469922 PMC3799810

[B60] Rana S. Dringen R. (2007). Gap junction hemichannel-mediated release of glutathione from cultured rat astrocytes. *Neurosci. Lett.* 415 45–48. 10.1016/j.neulet.2006.12.043 17222973

[B61] Ransom B. R. Giaume C. (2013). “Gap junctions, hemichannels,” in *Neuroglia*, 3rd Edn, eds Kettenmann H. Ransom B. R. (Oxford: Oxford University Press), 292–305.

[B62] Retamal M. A. Froger N. Palacios-Prado N. Ezan P. Sáez P. J. Sáez J. C. (2007). Cx43 Hemichannels and Gap Junction Channels in astrocytes are regulated oppositely by proinflammatory cytokines released from activate microglia. *J. Neurosci.* 27 13781–13792. 10.1523/JNEUROSCI.2042-07.2007 18077690 PMC6673621

[B63] Sanacora G. Banasr M. (2013). From pathophysiology to novel antidepressant drugs: glial contributions to the pathology and treatment of mood disorders. *Biol. Psychiatry* 73 1172–1179. 10.1016/j.biopsych.2013.03.032 23726152 PMC3688253

[B64] Seidel A. Arolt V. Hunstiger M. Rink L. Behnisch A. Kirchner H. (1995). Cytokine production and serum proteins in depression. *Scand. J. Immunol.* 41 534–538. 10.1111/j.1365-3083.1995.tb03604.x 7539545

[B65] Sluzewska A. Rybakowski J. Bosmans E. Sobieska M. Berghmans R. Maes M. (1996). Indicators of immune activation in major depression. *Psychiatry Res.* 64 161–167. 10.1016/S0165-1781(96)02783-7 8944394

[B66] Stehberg J. Moraga-Amaro R. Salazar C. Becerra A. Echeverría C. Orellana J. A. (2012). Release of gliotransmitters through astroglial connexin 43 hemichannels is necessary for fear memory consolidation in the basolateral amygdala. *FASEB J.* 26 3649–3657. 10.1096/fj.11-198416 22665389

[B67] Sun J. D. Liu Y. Yuan Y. H. Li J. Chen N. H. (2012). Gap junction dysfunction in the prefrontal cortex induces depressive-like behaviors in rats. *Neuropsychopharmacology* 37 1305–1320. 10.1038/npp.2011.319 22189291 PMC3306892

[B68] Tai Y. H. Tsai R. Y. Lin S. L. Yeh C. C. Wang J. J. Tao P. L. (2009). Amitriptyline suppresses neuroinflammation-dependent interleukin-10-p38 mitogen-activated protein kinase-heme oxygenase-1 signaling pathway in chronic morphine-infused rats. *Anesthesiology* 110 1379–1389. 10.1097/ALN.0b013e31819fccd5 19417613

[B69] Takeuchi H. Suzumura A. (2014). Gap junctions and hemichannels composed of connexins: potential therapeutic targets for neurodegenerative diseases. *Front. Cell. Neurosci.* 8:189 10.3389/fncel.2014.00189 PMC4151093 25228858

[B70] Tomaz V. S. Cordeiro R. C. Costa A. M. de Lucena D. F. Nobre Júnior H. V. de Sousa F. C. (2014). Antidepressant-like effect of nitric oxide synthase inhibitors and sildenafil against lipopolysaccharide-induced depressive-like behavior in mice. *Neuroscience* 268 236–246. 10.1016/j.neuroscience.2014.03.025 24662848

[B71] Valera E. Ubhi K. Mante M. Rockenstein E. Masliah E. (2014). Antidepressants reduce neuroinflammatory responses and astroglial alpha-synuclein accumulation in a transgenic mouse model of multiple system atrophy. *Glia* 62 317–337. 10.1002/glia.22610 24310907 PMC4183229

[B72] Vardjan N. Verkhratsky A. Zorec R. (2015). Pathologic potential of astrocytic vesicle traffic: new targets to treat neurologic diseases? *Cell Transplant.* 24 599–612. 10.3727/096368915X687750 25807491

[B73] Verkhratsky A. Rodríguez J. J. Parpura V. (2012). Neurotransmitters and integration in neuronal-astroglial networks. *Neurochem. Res.* 37 2326–2338. 10.1007/s11064-012-0765-6 22476701

[B74] Ye B. Shen H. Zhang J. Zhu Y. G. Ransom B. R. Chen X. C. (2015). Dual pathways mediate β-amyloid stimulated glutathione release from astrocytes. *Glia* 63 2208–2219. 10.1002/glia.22886 26200696

[B75] Ye Z. C. Wyeth M. S. Baltan-Tekkok S. Ransom B. R. (2003). Functional hemichannels in astrocytes: a novel mechanism of glutamate release. *J. Neurosci.* 23 3588–3596. 12736329 10.1523/JNEUROSCI.23-09-03588.2003 PMC6742182

[B76] Yirmiya R. Pollak Y. Barak O. Avitsur R. Ovadia H. Bette M. (2001). Effects of antidepressant drugs on the behavioral and physiological responses to lipopolysaccharide (LPS) in rodents. *Neuropsychopharmacology* 24 531–544. 10.1016/S0893-133X(00)00226-8 11282253

